# Individual level peer interventions for gay and bisexual men who have sex with men between 2000 and 2020: A scoping review

**DOI:** 10.1371/journal.pone.0270649

**Published:** 2022-07-15

**Authors:** Jack Freestone, Krista Joy Siefried, Garrett Prestage, Mohamed Hammoud, Angus Molyneux, Adam Bourne

**Affiliations:** 1 The Kirby Institute, University of New South Wales, Sydney, Australia; 2 ACON, Surry Hills, Australia; 3 The National Centre for Clinical Research on Emerging Drugs, c/o the University of New South Wales, Sydney, Australia; 4 Alcohol and Drug Service, St Vincent’s Hospital Sydney, Darlinghurst, Australia; 5 The National Drug and Alcohol Research Centre [NDARC], the University of New South Wales, Sydney, Australia; 6 Australian Research Centre for Sex Health and Society, La Trobe, Melbourne, Australia; Fairfield University Marion Peckham Egan School Of Nursing & Health Studies, UNITED STATES

## Abstract

**Background:**

Peer-led interventions are central to the global HIV response for gay and bisexual men who have sex with men [GBMSM]. Since the year 2000, technological advancements in HIV and an increased response to the health disparities faced by GBMSM outside of HIV, have contributed to the expanding scope of their content and delivery. This review sets out to characterise the evidence base for individual level peer interventions for GBMSM, overview approaches to implementing and evaluating them and identify future priorities for their delivery and evaluation.

**Methods:**

A scoping review methodology was applied and evaluations of peer programs for GBMSM published in peer reviewed journals were identified via subject heading and keyword searches across five electronic databases. Titles and abstracts were reviewed, and full texts were assessed against eligibility criteria. A coding framework was used to extract data from included studies against intervention implementation and evaluation components.

**Results:**

A total of 38 studies evaluating peer led interventions against effectiveness outcomes were deemed eligible for inclusion and coded into four intervention modalities; peer counselling [n = 6], groupwork programs [n = 15], peer navigation [n = 7] and peer education [n = 10]. Most addressed HIV [n = 32] and across intervention modalities, evaluations demonstrated compelling evidence of significant effect. Intervention effects on broader indicators of psychosocial wellbeing were not extensively evaluated. Expertise regarding the implementation and evaluation of peer interventions addressing HIV among GBMSM ought to be leveraged to expand the scope of peer intervention to meet the diverse health and wellbeing needs of GBMSM.

## Introduction

Peer interventions have been implemented across populations and domains of health and wellbeing for decades [1, 2]. They are central to the global HIV response for gay, bisexual and other men who have sex with men [GBMSM] [3, 4]. Prior studies and systematic reviews of peer interventions for GBMSM have included community level interventions and focus exclusively on outcomes related to HIV [5, 6]. Community level peer interventions for GBMSM seeking to influence widespread changes in social norms, attitudes, or behaviours have been evaluated in cross sectional or city comparison studies in several contexts globally and the literature broadly indicates that these interventions are efficacious in facilitating a reduction in HIV transmission risk behaviours [5, 7–9]. Implementation studies have also documented the acceptability, feasibility, and efficacy of engaging peers to promote and deliver HIV screening services for GBMSM [10–15]. Peer-led strategies to disseminate, promote and deliver HIV testing services have been favorably evaluated in terms of service level reach, access, cost effectiveness and HIV case finding [6, 16–20]. No reviews, to our knowledge, have focused on individual level peer interventions for GBMSM and assessed their effect on outcomes across broad domains of health and wellbeing.

Peer interventions for GBMSM commenced at the outset of the early HIV epidemic [21–23]. In the face of increasing violence and discrimination, community leaders leveraged traditions of care established throughout the gay and lesbian rights movement of the 1960s and 1970s and established activist groups, urgent HIV prevention education initiatives and support programs to assist those living with HIV and dying from AIDS [21, 22]. These early mobilisations around peer-based support were later formalised into structured peer-led responses and entered the realm of academic evaluation in the early 1990s. Early research on peer interventions were largely implemented and evaluated at the whole of community level and focused on Rogers’ diffusion of innovations’ theory [24] and typically trained popular opinion leaders to promote safe sex in community contexts to diminish community wide HIV risk behaviours [25, 26]. Since the year 2000 medical advancements in antiretroviral therapy that altered the treatment, management of HIV [27] gave rise to the development of peer interventions to support GBMSM to manage HIV at the individual level [28]. Since 2000 the development of mobile technology, hook up applications and social media that re-shaped the social structures of gay male communities [29] has led to peer interventions being implemented via text message or online settings [30–32].

Additional to peer interventions to support individual HIV management, it has been suggested that peer interventions for GBMSM may be used to help GBMSM manage health conditions outside of HIV [33, 34]. Post 2000 there has been an increasing acknowledgement that GBMSM face health and wellbeing disparities outside of HIV. A detailed summary of the varied health disparities experienced by heterogenous GBMSM globally is out of scope for this review however recent cross-sectional and longitudinal cohort studies among GBMSM have observed health inequalities relating to the experience of anxiety, depression, or suicidal ideation [35–38]. Several studies and systematic reviews reflect a practice of sexualised drug use among GBMSM and observe sexual risk taking, poor mental health and psychosocial outcomes among their participant samples; noting complex relationships between sexualised drug use and health outcomes [38–42]. Peer led service based responses addressing these mental health and substance use disparities often prioritise intervention modalities that can be tailored to individual experiences [43].

As prior reviews have included community level interventions and focused on HIV [5, 44] and given that GBMSM face health disparities additional to HIV that may be addressed by peer interventions delivered at the individual level, a scoping review of the contemporary evidence base for individual level peer interventions for GBMSM across domains of health and wellbeing is warranted. We define an individual level intervention in accordance with the social ecological model of health as any intervention delivered to support intrapersonal knowledge, attitudes or behaviours [45] and we include studies that measure outcomes at the individual level. We define peer interventions in alignment with the formative and frequently cited framework articulated by Simoni et al who propose four definitional components for peer interventions. *[1]* Peers share key personal characteristics, circumstances, or experiences with those for whom they provide interventions. *[2]* They are valued and are effective because of their peer status. *[3]* They lack formal professional training or qualifications and *[4]* they deliver health interventions intentionally according to a set of standard protocols rather than operating as part of a naturally occurring social network [1].

In alignment with a scoping review methodology outlined by Arksey and O’Malley [46], this review sets out to characterise the evidence base for individual level peer interventions and overview approaches to implementing, and evaluating them. It aims to identify future areas for intervention implementation and evaluation and summarise learning from the evidence base to guide future practice.

## Methods

### Search

**Fig 1:**
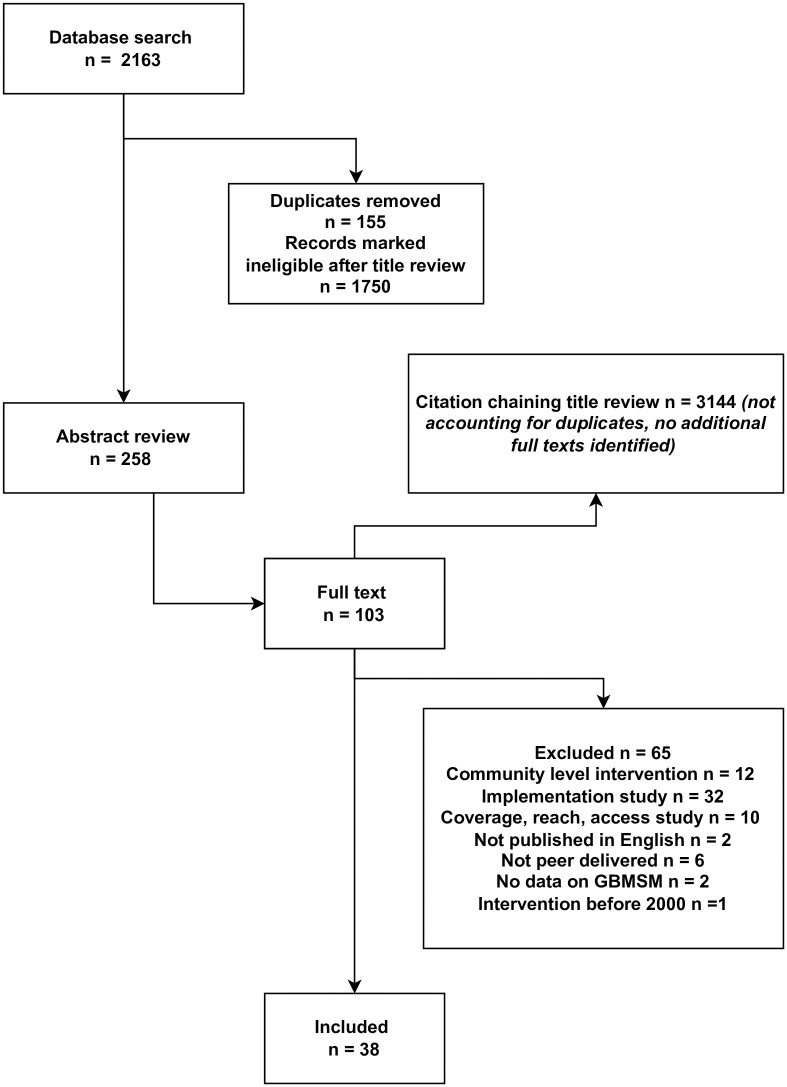
Study selection flowchart.

A search strategy was devised in collaboration with a librarian and five electronic databases were searched (MEDLINE, Scopus, Embase, CINAHL and PsycINFO). Search terms were constructed using key words and relevant subject headings. To ensure broader health issues were captured, search terms related to HIV were not part of the search strategy. Searches within the title, abstract or keywords across all databases comprised of 3 concepts. [1] Peers, including the terms “peer education”, “peer support”, “peer program”, “social support”, “lay health advisor”, “peer navigation”, “peer to peer” and “peer group”. [2] Gay and bisexual men who have sex with men, including the terms “gay”, “bisexual”, “MSM”, “men who have sex with men” and “homosexual male”. [3] Programs, including the terms “programs”, “evaluation”, “intervention”, and “effectiveness”. The search was limited to studies published between January 2000 and December 2020. The first author also reviewed the bibliographies of all finally included studies and used Google Scholar for reverse citation chaining to review the titles of all articles that cited one of the 103 papers included at the stage of full text review [Fig 1]. The identification of literature was conducted by the first author, with oversight by and consultation with co-authors who provided initial guidance on inclusion and exclusion criteria and ongoing support with eligibility screening. Endnote was used as data management tool.

### Inclusion

Included studies evaluated interventions that: [1] were delivered by peers who functioned according to standardised protocols, [2] where peers shared lived experience or identity characteristics with intervention recipients and [3] were delivered to support intrapersonal knowledge, attitudes or behaviours, [4] targeted GBMSM [or synonymous terms: gay men, MSM, bisexual men] and [5] were implemented between the year 2000 and 2020. To be included evaluations of these interventions needed to follow the same individuals over time and report effect sizes on outcomes relating to behaviour change, knowledge beliefs and attitudes, service access, HIV or STI incidence, mental health, psychosocial wellbeing, or biomarkers relating to either HIV, or AOD outcomes. Studies needed to be published in English, in peer reviewed journals, between 2000 and 2020.

Included in this review are several social network interventions [47–51]. Although designed for a community level effect, these are included because their delivery supported intrapersonal knowledge attitudes and behaviours and they used prospective designs to measure changes in outcomes among individuals.

### Exclusion

Studies that evaluated the community level effect of an intervention were excluded. Studies were also excluded if they did not define peers based on identity or lived experience characteristics. Studies with mixed samples, where GBMSM participants were not the majority were excluded. Studies evaluating program implementation in terms of acceptability, feasibility, access, coverage, and cost effectiveness were excluded, note these were largely evaluations of peer led HIV screening interventions.

### Coding

Previous systematic reviews informed a data coding framework for this scoping review [5, 44, 52]. A coding framework was devised by all authors and this framework stipulated data extraction in accordance with both intervention and evaluation components. Data from interventions were extracted in relation to country of implementation, target population, intervention setting, the theoretical models on which interventions were based and intervention type. Data from full texts were extracted by the first author and reviewed by co-authors for both study eligibility and extraction accuracy.

As outlined in Table 1, interventions were categorised into four types and defined in alignment with previously published literature reviews on peer programs and practice guidelines for peer program practitioners [3] and shaped by the first author’s analysis of intervention descriptions. As such, *Peer counselling* interventions were defined as individually tailored cognitive behavioural or motivational talking therapies delivered to manualised protocols. *Groupwork programs* encompassed peer-led group facilitation or group based mutual sharing, discussion, and structured learning. *Peer navigation* interventions were defined as ongoing programs to support participants with access to services, or adherence to primary care. *Peer education* interventions were defined as activities encompassing brief information sharing or health promotion often taking place in naturally occurring social settings. Evaluation data were extracted as per study type, primary outcomes, sample size, follow up time, retention, effect size and a description of methodological limitations.

**Table 1:** Included studies by intervention type, region, year of publication, study type and health domain.

	Counselling (n = 6)	Groupwork (n = 15)	Navigation (n = 7)	Education (n = 10)
**Region**				
North America (n = 22)	5	8	5	4
Asia (n = 5)	1	1	1	2
Australia (n = 3)	0	3	0	0
Africa (n = 2)	0	1	1	0
Europe (n = 5)	0	2	0	3
South America (n = 1)	0	0	0	1
**Year**				
2015–2020 (n = 22)	2	8	6	6
2010–2014 (n = 9)	3	3	1	2
2005–2009 (n = 5)	1	3	0	1
2000–2004 (n = 2)	0	1	0	1
**Study type**				
RCT (n = 20)	4	6	3	7
Quasi experimental (n = 1)	0	0	0	1
Pre-post cohort (n = 17)	2	9	4	2
**Intervention health domain**				
HIV (n = 32)	6	10	7	9
Substance use (n = 5)	0	4	0	1
Body image (n = 1)	0	1	0	0

This scoping review did not involve primary research with human subjects and therefore did not warrant institutional ethics approval.

## Results

Database searches turned up 2163 articles [155 duplicates] for title review. Of these, 258 were selected for abstract review and 103 full texts were reviewed, with their bibliographies read and reverse citations searched on Google Scholar to identity further relevant studies. A total of 3144 titles [not accounting for duplicates] were reviewed throughout Google Scholar citation chaining. A total of 38 studies were finally included.

Of the 38 included studies most were from North America (n = 22) and published between 2015 and 2020 (n = 15). Most studies were either RCT’s (n = 20) or single arm pre-post cohort studies (n = 17), Interventions were primarily delivered to address HIV outcomes (n = 32), with five interventions addressing substance use and one addressing body image. Of the six interventions without a primary focus on HIV, five were groupwork programs. As outlined in Table 2 interventions commonly targeted HIV positive GBMSM. Nearly half of the 21 interventions from the USA were tailored for African American or Latinx GBMSM (n = 8). Peer interventionists were defined as peers in most studies because of their status as GBMSM, other shared identity and lived experience characteristics pertaining to HIV status and race, with other lived experiences relating to substance use or PrEP use less frequently prioritised.

**Table 2:** Included studies by target sub populations and peer identity characteristics.

	Counselling (n = 6)	Groupwork (n = 15)	Navigation (n = 7)	Education (n = 10)
**Target sub populations** *				
GBMSM (no sub population)	0	5	0	4
HIV + status	3	4	6	0
HIV negative or status unknown	0	2	1	4
Trans women (alongside GBMSM)	0	1	4	1
Young (age range 16–34)	0	2	1	0
GBMSM reporting sex with women	2	0	0	0
Racial group**	1	3	1	3
**Peer identity characteristics** ***				
GBMSM	4	13	4	9
HIV + status	3	5	2	0
Race	3	2	2	4
Substance use	0	1	1	1
Other lived experience****	0	0	3	0

*Interventions list multiple target sub populations and are counted more than once

** Among interventions delivered in the US (n = 21), 8 targeted ethnic minorities: African American GBMSM (n = 4), Latnix GBMSM (n = 3) or both African American and LATINX GBMSM (n = 1)

***Interventions that stipulate multiple identity criteria for peers are counted more than once

**** Other lived experiences related to HIV treatment experience, PrEP use, histories of incarceration and family or social network participation

### Peer counselling

Six evaluations of peer counselling interventions were identified [53–58] (S1 Table), all were based on the information, motivation and behavioural model [59]. Five followed individually tailored one-to-one concurrent session protocols [53–56] and one included a single session motivational interview [58]. All multiple session interventions used flexible manualised protocols to ensure peers delivered essential intervention information and therapeutic processes while enabling participants to direct sessions as per their personal needs and circumstances [53–57].

Many authors noted mental health and substance use concerns among their participant samples [53–57]. Apart from one intervention that exclusively addressed HIV risk and screening [58], the remaining included content addressing mental health, relationships, identity, self-esteem and substance use [53–57] and four included educational components focused on intimacy, relationships, communication, and coping skills [53, 55–57]. However, overall peer counselling interventions prioritised HIV and intervention components addressing psychosocial wellbeing were delivered with the intention of bolstering resilience to mediate HIV risk, rather than to bolster wellbeing as an independent end goal.

Peer counselling interventions described resource intensive training and supervision support processes for peer interventionists. They described multiple day training programs covering motivational interviewing, inclusivity, non-judgmental communication, and empathy. Three studies reported either individual or group based weekly supervision meetings intended to support peers to workshop challenges, develop skills and monitor for personal safety and wellbeing [55–57]. Four studies acknowledged the importance of monitoring peer delivery with intervention fidelity measures [54, 55, 57, 58] and three included fidelity monitoring processes to continually build capacity among peer interventionists [55, 57, 58].

#### Evaluation

Five peer counselling interventions assessed HIV risk behaviours as their primary outcome [53–57] of these two RCT’s [54, 55] and two cohort studies [56, 57], demonstrated evidence of significant positive effect. One RCT that failed to report a significant effect on HIV risk behaviours, compared a four-session peer counselling intervention to a culturally tailored single session HIV testing and counselling intervention which proved efficacious, reducing the ability to detect significance within the experimental arm [53]. In two studies, effect sizes associated with peer intervention were reportedly reduced due to the high standard of routine HIV primary care provided across both experimental and control conditions [54, 55].

Although five interventions addressed substance use, only three studies presented data to indicate that interventions were associated with reductions in substance use, two reported reductions of substance use prior to sex [54, 56], two reported reductions in overall substance use [54, 55] and one analysed substance use as a mediator of HIV risk [55]. In only reporting substance use outcomes relating to use reduction and HIV risk, it appears interventions prioritised abstinence for the sake of HIV prevention rather than focusing on AOD harm reduction education, or linkage to AOD services. Only one peer counselling study [56] reported outcomes related to social support, self-esteem, and loneliness with no other studies reporting outcomes related to psychosocial wellbeing, Therefore, the ability of semi-structured peer counselling interventions to improve mental health outcomes among GBMSM remains unknown.

In reporting effect sizes, peer counselling studies only one study validated self-report data with the use of service attendance data [58]. Future evaluations of peer counselling interventions should endeavor to validate self-report with biomarkers or service level data when measuring intervention effects.

### Groupwork programs

Fifteen groupwork interventions were identified (S2 Table), apart from one intervention delivered online groupwork programs were universally delivered in community-based settings. The online program ran for seven weeks and comprised of information modules, action planning activities, moderated discussion boards and weekly peer facilitated live chats [30]. The use of community settings to host groupwork reflects the rationale that peer groupwork programs for marginal populations both rely on and enable community connection. Many evaluations appraised groups for their multiple benefits of bolstering knowledge and supporting healthy behaviours while increasing social support, self-esteem, and other markers of psychosocial wellbeing [51, 60–64].

Groupwork delivery formats spanned, consecutive day intensives [60, 64], one to four hour weekly sessions lasting between two to eight weeks [61–63, 65–72], online [30], or groupwork nested in a broader project or intervention implementation [51]. Programs combined information provision with collaborative activities, behavioural role plays and strategies akin to behavioural talk-based therapies. Like peer counselling, group programs were predominately founded on the information motivation and behavioural model [65–68], social cognitive theory [30, 60, 63–65, 68–70] and the transtheoretical model of behaviour change [60, 63, 64].

Apart from one program addressing body image among college aged GBMSM [71], group programs may be stratified by their primary subject matter: **[1]** Groups imparting HIV education and HIV testing service referrals [51, 60, 61, 63, 64, 69, 70], **[2]** groups supporting HIV management [30, 66, 68] and **[3]** groups addressing substance use delivered in combination with psychologists [65, 67], or nicotine replacement therapies [62, 72].

#### Evaluation

Nine evaluations of groupwork programs used pre-post single arm cohort study designs and reported evidence of significant positive effect against at least one outcome [51, 60–61, 64–66, 70, 72], the remaining six were RCTs [30, 63, 67–69, 71]. One RCT comparing HIV risk behaviours and status disclosure outcomes among HIV positive GBMSM failed to find evidence of significant effect at 6 months follow up [68].

Despite short follow up times, only four group programs reported greater than 80% retention [62, 68, 69, 71], this may reflect a reality that groupwork interventions are susceptible to participant dropout, or it may reflect challenges associated with data collection at the community sites where group programs were often implemented [51, 61].

As with peer counselling interventions, evaluations of groupwork programs prioritised outcomes regarding HIV. Among programs for HIV negative GBMSM four reported significant effects on HIV risk behaviours [60, 63, 69, 70] and five reported significant effects on receipt of HIV testing [51, 63, 64, 69, 70]. Across four interventions addressing substance use outcomes, positive intervention effects were observed on abstinence from smoking [62, 72], modest reduction in methamphetamine use [65] and reduced number of days on which alcohol was consumed [67].

Despite the community connection and network formation rationale for groups, only one study specifically examined loneliness [66]. Loneliness is increasingly understood to mediate quality of life GBMSM [73] and whether peer-based group programs for GBMSM effectively bolster social connectedness and combat loneliness is unexplored in the literature.

Seven studies reported outcomes relating to psychosocial wellbeing [30, 51, 65, 66, 69, 71, 72] and positive intervention effects were observed against measures of body image, dietary restraint and bulimic symptoms [71], fear of being rejected [66], body change and relationships [30], fatalism [69], sexual identity acceptance [51], depression and anxiety [72] and psychological distress [30, 65].

Given a reliance on short follow up periods one cannot determine whether positive groupwork intervention effects on either HIV or psychosocial outcomes are sustained over time. The longest follow up time for any group evaluation was 12 months [67] with the remainder following participants for 6 months or less.

Overall, evidence in favour of groupwork programs is compelling, owing to their mode of delivery it would seem groups have the potential to reduce loneliness and bolster wellbeing. However, the long-term effects group interventions on loneliness and psychosocial wellbeing are not clearly determined by the published literature.

### Peer navigation

Seven peer navigation interventions were included in this review (S3 Table) [28, 32, 74–78]. Of these, six addressed HIV treatment and primary care engagement among HIV positive participants [28, 32, 74, 75, 77, 78] and one addressed PrEP access among HIV negative transgender women and GBMSM [76].

Peer navigation interventions observed that recipients faced multiple vulnerabilities across domains of health and wellbeing including mental health, housing, substance use, employment, and intimate partner violence. In recognition of these challenges, peer navigation programs were conceived as strategies to ensure retention in HIV care as a pathway for referrals outside of HIV [28, 74, 76, 79] or HIV oriented interventions where peers were also trained to support linkage to a broad array of health and wellbeing services [32, 75, 76].

The use of social media, webchat, and text message to support intervention communication and sustain engagement was ubiquitous among peer navigation interventions. Two interventions were exclusively digitally delivered [32, 77], one incorporated automated theory informed text messaging to support PrEP adherence [76] and the remaining included ad hoc digital communication between peer interventionists and participants [28, 74, 75, 78].

Although peer navigation interventions aimed to support recipients to adhere to medication regimes or appointment schedules in primary care, they also incorporated elements of psychoeducation and behavioural therapies. Five explicitly described their application of the information motivational behavioural model [28, 74, 76] or social cognitive theory [74–77] to support motivation, self-efficacy, and behaviour change. Interventions also prioritised the therapeutic importance and social support derived from the peer and participant relationship [28, 74, 77], highlighting that these interventions provide more than just ancillary support to primary care [75].

#### Evaluation

All evaluations reported positive effects in relation to care linkage, retention, and adherence outcomes. Significant effects were reported as they related to HIV viral load [28, 32, 77], HIV primary care attendance [74, 77] antiretroviral adherence [74] and linkage to PrEP [76] or HIV primary care [75, 78].

Most retained a singular focus on outcomes related to HIV care with only one RCT reporting significant positive intervention effects associated with access to community based mental health services [75]. Peers were positioned to assist participants to navigate multiple domains of health and wellbeing and a singular focus on HIV outcomes represents a limitation in the scope of peer navigation evaluations.

All peer navigation studies for HIV positive participants recruited their sample from primary care with only two specifying prior poor retention in care in their eligibility criteria [32, 80], potentially introducing a ceiling effect [74]. However, because peer navigation interventions were associated with primary care, they were less likely to only collect self-reported data than other intervention types. Four evaluations reported HIV viral load testing [28, 74, 75, 77] and five reported service monitoring data [28, 74, 75, 77, 78]. Two studies that reported significant positive effects on HIV viral load [28, 75] failed to find significant effect on self-reported ART adherence, with high levels of adherence reported in both arms, potentially signifying recall and social desirability biases associated with self-report.

Use of service monitoring data and biomarkers represents a strength of peer navigation evaluations, however in general across evaluations the prioritisation of clinical measurement came at the expense of collecting self-reported data to examine broader health and wellbeing. While the literature clearly demonstrates that peer navigation interventions for GBMSM are effective in relation to linkage, retention, and adherence to HIV care, whether their benefits extend beyond these outcomes is undetermined.

### Peer education

A total of 10 peer education interventions that met inclusion criteria were included (S4 Table) [31, 47–50, 81–85]. Nine described processes where peer leaders completed group training programs and learnt to impart theory based HIV prevention messages or promote HIV screening online [31, 84, 85], in person [47–49, 83, 86] or across a combination of online and in person [50] social settings. Only, one intervention addressing methamphetamine use and HIV risk behaviours in the context of methamphetamine use, followed a different mode of delivery. In this intervention peer education occurred via interactive texts alongside digital intervention components including automated text messages and online behavioural self-assessments [82]. Online peer education interventions typically leveraged private group and private chat functions on popular social media sites to enable ongoing communication between peer educators and peers for the duration of the intervention [31, 84, 85].

Nine peer education interventions were underpinned by either the diffusion of innovations [24] or social cognitive theory [87]. These theories emphasize positive role modelling and influence as such peer education interventions were more likely than other intervention types to account for the ways group level norms influence the attitudes and behaviours of individuals. Peer educators were therefore positioned as responsible for modelling the positive health behaviours and attitudes prioritised by interventions. As such, peer education interventions were more likely to invest in robust processes around peer recruitment than other intervention types. Most described the selection and appraisal of peers against criteria of being community connected, well respected, enthusiastic, effective communicators with an ability and willingness to adhere to intervention and study procedures. Peers were identified and recruited by a variety of processes, including ethnographic observation [50, 81], sociometric mapping to identity network centrality [47–49], referral from community-based organisations [31, 81, 84, 85] or word of mouth [83].

#### Evaluation

The ten included interventions demonstrate strong evidence that peer education positively impacts mediators of behaviour change [31, 47–50, 81, 83]. Interventions were associated with significant improvements in HIV [47, 48, 83] and PrEP [50] related knowledge, increased intentions to practice HIV risk reduction [47–49], test for HIV [31] or start using PrEP [50] condom use skills [83] peer norms and self-efficacy related to safer sex [47–49] and PrEP use [50].

Reports of significant effect in relation to behaviour change were mostly limited to increases in condom use [47–49, 82, 83] or uptake of HIV testing [83–85] or PrEP [31]. With only one intervention examining and reporting significant effects regarding days of methamphetamine use [82].

Of peer education studies, one study corroborated self-reported data with tests for incident HIV or STIs [49], another two verified data by analysing service access or requests and returns of HIV self-testing kits [84, 85], the remaining studies collected self-reported data exclusively. Strategies to reduce recall and social desirability biases associated with self-report included the use of short recall periods [47, 83], anonymity [31], self-administered surveys and audio computer assisted surveys [82].

As peer interventions were described as non-manualised, brief, or informal interventions, they are possibly more susceptible to effect attenuation by comparison to longer and multifaceted peer interventions. Among studies included in this review only four reported follow-up periods of longer than 6 months [47, 49, 82, 83], of these three reported data from multiple follow up points [47, 49, 82] and two reported effect attenuation regarding HIV risk behaviours [47, 49]. Given the potential susceptibility of peer education interventions to effect attenuation, future studies that measure outcomes over multiple follow-ups and longer follow up periods ought to be prioritised.

## Discussion

The studies included in this review demonstrate that peer interventions for GBMSM are broadly effective when evaluated against outcomes related to HIV. Across four intervention types, peer interventionists were appraised for their ability to facilitate access to meaningful and effective interventions. It has been observed that GBMSM may experience barriers to accessing traditional modalities of healthcare, owing to experiences of stigma and discrimination and the perception that mainstream healthcare services lack sufficient cultural awareness and expertise to meet the needs of GBMSM [88]. As is reflected in our findings for groupwork programs, peer interventions provide a valuable space for GBMSM to network and connect on lived experiences as they relate to sexual orientation and a range of other identity characteristics. Many peers providing interventions in our included studies reflected the identities of their intervention recipients on multiple axes, highlighting how peer programs may be tailored to meet the needs of GBMSM with multiple and intersecting identities [89].

Our findings demonstrate that between 2000 and 2020, peer interventions have targeted GBMSM who are HIV positive, HIV negative, Latinx, African American or young GBMSM aged below 34. Six interventions were delivered for trans women alongside GBMSM, no interventions were delivered specifically for trans men, and no studies reported disaggregated findings for cisgender and transgender GBMSM. A recent study comparing trans GBMSM to cisgender GBMSM found that trans GBMSM are more likely to report experiences of discrimination in the health care system and more likely to self-rate their health as poor [90]. Given these disparities peer interventions tailored for trans GBMSM ought to be prioritised and data regarding outcomes for trans GBMSM engaged in peer interventions ought to be sufficiently collected and reported. This review included seven studies where peer delivered interventions were exclusively online or via text message. All virtual interventions demonstrated evidence of significant effect and were published within the last ten years. In addition to interventions that were exclusively virtual were several interventions that incorporated components of digital delivery and these components were appraised to support participant learning or peer to participant rapport and relationship building. While other reviews have demonstrated that digital health interventions to support HIV prevention among GBMSM are feasible and acceptable [91], our review demonstrates that contemporary peer interventions for GBMSM are reflecting contemporary social structures of GBMSM communities and effectively incorporating digital communications.

This review has observed that many evaluations of peer interventions assessed HIV oriented outcomes to the exclusion of evaluating impacts on broader health and wellbeing. This scope is understandable; GBMSM face pronounced disparities with regards to HIV and peer interventions for GBMSM are most often conceived and funded to address HIV specifically. Notwithstanding concern for HIV, authors of included studies noted high rates of psychosocial distress and substance use among their participant samples, mirroring the broader evidence base relating to the health of GBMSM [38, 42, 92, 93]. As a result, peer counselling, groupwork and navigation interventions were likely to describe the inclusion of content addressing mental health, substance use, relationships, or access to social support services, although outcomes related to these topics were often not comprehensively evaluated. Further, evaluations of groupwork and peer education programs were commonly limited by short follow up periods, while peer counselling interventions often exclusively relied on self-reported data and peer navigation interventions reported data from primary care at the expense of self-reported data relating to psychosocial wellbeing.

Studies that collected data on substance use examined outcomes exclusively in relation to use reduction or HIV risk behaviour. While these outcomes are important, peer interventions that address substance use have the potential to effect a range of other outcomes such as harm reduction knowledge and practice, overdose experience, emergency admission, hepatitis C transmission or AOD service access. These outcomes were not widely examined. Substance use is singled out here because of the long standing and comprehensive evidence base indicating that GBMSM use substances at high rates, experience unique harms related to use and report barriers to accessing traditional or mainstream AOD services [94, 95]. As such the needs of GBMSM in relation to substance use may be well met by peer programs, however from the current evidence base one cannot determine whether peer interventions addressing substance use among GBMSM are truly effective. Going forward, practitioners in community health and research, must consider how evaluations of existing peer interventions for GBMSM can better measure a broader range of their impacts and endeavor to comprehensively assess outcomes as they relate psychosocial wellbeing. There is also an imperative to develop more peer interventions for GBMSM that specifically address health areas outside of HIV. In developing and evaluating such interventions, practitioners must leverage the models for implementing, and evaluating peer interventions that have been generated throughout decades of a peer-based response to HIV among GBMSM. There are established protocols around the recruitment, training and supervision of peers and models for the evaluation of a diverse array of peer interventions[1, 3]. With compelling evidence to indicate that peer interventions can be effective, it is time to leverage expertise regarding their implementation and evaluation and further expand their scope to meet the diverse needs of GBMSM.

### Limitations

The search strategy for this scoping review did not extend to grey literature. Our intention was to focus on formal academic evaluation of peer interventions, however we acknowledge that some peer interventions for GBMSM may not be published in peer reviewed journals. As such, some examples of promising practice may not be reflected in this review. Two papers were excluded because they were not published in English. This review was structured by intervention type to increase its utility for practitioners working in alignment with pre-defined intervention modalities. However, this structure may mask the versatility and flexibility of peers who often work across several intervention modes of delivery simultaneously. In some circumstances interventions were challenging to code to specific intervention types, these were interventions where peers occasionally operated across modes of delivery within multifaceted and multilayered interventions, While we have acknowledged where peer programs are nested within multifaceted interventions [51, 82], we also acknowledge that peers do not always work to rigid protocols and coding some interventions by intervention type may be limiting.

## Supporting information

S1 Table Peer counselling. (DOCX)

S2 Table Groupwork programs. (DOCX)

S3 Table Peer navigation. (DOCX)

S4 Table Peer education. (DOCX)

S1 Checklist Preferred Reporting Items for Systematic reviews and Meta-Analyses extension for Scoping Reviews (PRISMA-ScR) checklist. (DOCX)
