# Supplementary material for: Individual level peer interventions for gay and bisexual men who have sex with men between 2000 and 2020: A scoping review
Source: PLoS One. 2022 Jul 15;17(7):e0270649. doi: 10.1371/journal.pone.0270649 (PMC9286286; doi:10.1371/journal.pone.0270649)
Supplement: S1 Table — (DOCX) [file pone.0270649.s001.docx]

| **Supplementary Table A: Peer counselling** | | | | | | | | | |
| --- | --- | --- | --- | --- | --- | --- | --- | --- | --- |
| **Ref #** | **Author**  **Year**  **County** | **Population** | **Intervention** | **Peer Identity Characteristics** | **Comparison** | **Study Type** | **Primary Outcomes** | **Sample**  **Follow Up**  **Retention** | **Effect Description** |
| 53 | Arnold  2019  USA | GBMSM who also have sex with women | Peer led 4 session motivational interview, HIV testing and counselling. | Race | Peer led HIV testing | RCT | HIV risk behaviours | N = 396  9 months  81% | No significant differences between intervention and comparison, both conditions reporting significant reductions in condomless sex with casual partners |
| 54 | Liu  2018  China | GBMSM, HIV positive | Peer led 4 session, 60 minute one to one counselling, focusing on HIV risk behaviours, HIV management and substance use. | HIV +, Sexuality | Standard of care for newly diagnosed HIV | RCT | HIV risk behaviours, substance use | N = 367  1 year  85% | Observed a 14 to 43% decrease in the prevalence of selected high-risk behaviours after HIV diagnosis. Peer counselling had a greater impact in reducing condomless anal sex with men, illicit drug use and condomless vaginal sex with women over time. |
| 57 | Safren  2011  USA | GBMSM, HIV positive | 5 session peer delivered counselling session, manualised intervention with tailored components addressing HIV risk behaviours, treatment adherence, drug use, stress management, relationships, triggers, and HIV disclosure. | HIV +, Sexuality | NA | Pre post cohort | HIV risk behaviours | N = 176  1 year  82% | No overall change in HIV risk behaviour for entire sample. Significant reductions in HIV risk behaviour among those reporting risk behaviours at baseline. |
| 56 | Operario  2010  USA | GBMSM who also have sex with women | Peer led 4 session motivational interview, HIV testing and counselling. | Race | NA | Pre post cohort | HIV risk behaviours, substance use, psychosocial wellbeing | N = 68  3 months  53% | Significant reductions in unprotected receptive and insertive anal sex with male partners, fewer numbers of unprotected sex partners, decreased sex while on drugs. Increases in social support, self-esteem, and reduced loneliness. |
| 58 | Outlaw  2010  USA | GBMSM, African American | 30-minute intervention with a peer outreach worker in a community outreach venue. HIV oriented peer education and safe sex supplies offered plus a motivational interview addressing HIV testing and counselling offered. | Age, Race, Sexuality | Peer education in an outreach setting, HIV education and HIV testing, and counselling offered. | RCT | Service access | N = 188  NA  100% | Almost half (49%) of participants in the intervention group received HIV counselling and testing vs. 20% of the participants in control. Intervention participants more likely to return for test results (98% vs. 72%). |
| 55 | McKirknan  2007  USA | GBMSM, HIV positive | Peer led 4 session, one to one counseling intervention focusing on HIV risk behaviours, HIV management, psychosocial wellbeing, and substance use | HIV +, Sexuality | Standard of care | RCT | HIV risk behaviours | N = 313  1 year  80% | Transmission risk among intervention participants decreased from 34% at to 20% at both 6 and 12 months. Transmission risk remained constant in comparison arm |
