# Supplementary material for: Individual level peer interventions for gay and bisexual men who have sex with men between 2000 and 2020: A scoping review
Source: PLoS One. 2022 Jul 15;17(7):e0270649. doi: 10.1371/journal.pone.0270649 (PMC9286286; doi:10.1371/journal.pone.0270649)
Supplement: S2 Table — (DOCX) [file pone.0270649.s002.docx]

| **Supplementary Table B: Groupwork programs** | | | | | | | | | |
| --- | --- | --- | --- | --- | --- | --- | --- | --- | --- |
| **Ref #** | **Author**  **Year**  **County** | **Population** | **Intervention** | **Peer Identity Characteristics** | **Comparison** | **Study Type** | **Primary Outcome Domains** | **Sample**  **Follow Up**  **Retention** | **Effect Description** |
| 64 | Abubakari  2020  Ghana | GBMSM, HIV negative or status unknown | Weekend peer education retreat, focusing on HIV prevention, based on US Many Men Many Voices intervention. | Sexuality | NA | Pre post | HIV risk behaviour, service access | N = 57  1 week  65% | Observed increases in HIV testing (from 4% to 17%) and increases in the relative frequency of condom use for anal, oral, and vaginal sex although this difference was not reported to be significant. |
| 65 | Burgess  2018  Australia | GBMSM | Two phase group program from methamphetamine dependence. Six-week clinician led psychoeducation, CBT, and motivational interviewing group intervention plus peer facilitated booster program as an open and ongoing group. | HIV +, Sexuality, Substance use | NA | Pre post | Substance use, knowledge, and attitudes psychosocial wellbeing | N = 55  1 month  58% | Modest improvements in participant psychological distress, personal well-being and stage of change plus reductions in methamphetamine use post intervention. |
| 69 | Rhodes  2017  USA | Latinx GBMSM & transgender women | Peer facilitated groupwork program delivered over four afternoon sessions, four hours each, addressing HIV/STI knowledge and self-efficacy. | Race, Sexuality | Four sessions of peer delivered general health care education. | RCT | HIV risk behaviours, service access, knowledge attitudes, psychosocial wellbeing | N = 304  6 months  100% | By comparison to control, intervention participants reported significantly improved outcomes related to consistent condom use in previous 3 months Adjusted Odds Ratio = 4.1  (95% CI = 2.2 – 7.9) and HIV testing during the past 6 months, Adjusted Odds Ratio=13.8 (95% CI = 7.6 = 25.3). |
| 51 | Shelley  2017  USA | GBMSM aged 18 – 29 | ‘Mpowerment’ intervention designed to reduce risky sexual behavior and increase HIV testing. Multi-faceted community-level intervention addressing psychosocial factors, includes a core group, outreach, M-Groups, publicity, and a project space (drop in). | Age, Sexuality | NA | Pre post | HIV risk behaviours, knowledge attitudes, psychosocial wellbeing | N = 436  6 months  60% | HIV testing increases from 53.6% at baseline to 70.2 at 6 months. Self-efficacy for safer sex increased at 3 month and 6 months follow up. No significant effects on HIV risk behaviours were observed at 6 months. |
| 66 | Hart  2016  Canada | GBMSM, HIV positive | Eight-week peer led groupwork program, information provision, motivational interviewing, and behavioural role plays, with a focus on HIV risk behaviours. | HIV +, Sexuality | NA | Pre post | HIV risk behaviours, psychosocial wellbeing | N = 82  3 months  72% | Significant reduction in condomless anal sex with HIV-negative and unknown HIV-status partners, from 50.0% at baseline to 28.9%. Significant reductions were also found in the two secondary psychosocial outcomes, loneliness, and sexual compulsivity. |
| 30 | Millard  2016  Australia | GBMSM, HIV positive | Seven-week online self-management group program comprised of information modules, action planning activities, moderated discussion boards and weekly peer facilitated live chats. Aimed to increase confidence, and abilities to manage psychological wellbeing. | HIV + | Usual HIV primary care | RCT | Psychosocial wellbeing, knowledge and attitudes | N = 132  3 months  61% | Significant improvement in the intervention group on a range of subscales assessing HIV quality of life, health education and self-efficacy. |
| 71 | Brown  2015  USA | GBMSM, college aged | Two session, peer led group, addressing gay community body image ideals, discussion, role plays, homework, and structured learning. | Age, Gender | Waitlist control | RCT | Psychosocial wellbeing | N = 87  4 weeks  86% | Significant decreases in body dissatisfaction, drive for muscularity, self-objectification, partner-objectification, body-ideal internalization, dietary restraint, and bulimic symptoms compared to waitlist control. |
| 60 | Stein  2015  USA | GBMSM, African American & Latinx | Peer led groupwork intervention based on Many Men ‘Many Voices’ protocol addressing HIV/STI risk & community building. Delivered over 6 sessions, as a weekend retreat or 1 week 3 session delivery. | Race, Sexuality  *Not stated in paper but in intervention protocol | NA | Pre post | HIV risk behaviours | N = 612  6 months  40% | Significant positive intervention effect observed in relation to each of the primary HIV risk behavioural outcomes. Number of sex events without a condom in the previous 3 months declined from a mean of 3.39 at baseline to 1.79 at follow up. |
| 72 | Dickson  2014  Switzerland | GBMSM | Nicotine replacement therapy and seven-week, peer led group work sessions, 2 hours each session. General population smoking cessation intervention repurposed for gay and bisexual men. | Sexuality | NA | Pre post | Substance use, psychosocial wellbeing | N = 70  6 months  54% | Significantly increased point prevalence of abstinence at 6 months. Participants who were lost to follow-up smoked more cigarettes and were more nicotine dependent at baseline than the participants who were retained. |
| 61 | Bavinton  2013  Australia | GBMSM | Six-week peer led group program, addressing HIV risk reduction, broader health and wellbeing, negotiation around safe sex and connection to queer communities | Sexuality | NA | Pre post | Knowledge and attitudes (sexual health) | N = 399  6 weeks  67% | Participants self-reported scores on sexual health capacity scale significantly increased after intervention. |
| 70 | Zhang  2010  China | GBMSM | Four 1.5-hour peer group sessions addressing HIV risk behaviours, with activities such as role playing, games, group discussions and brainstorming. | Sexuality | NA | Pre post | HIV risk behaviour, service access | N = 218  3 months  77% | Significant increases in self-reported condom use with another man (55.3% at baseline to 65.2% at follow up). HIV testing increased from 10.0% at baseline to 52.4% at follow up. |
| 63 | Wilton  2009  USA | GBMSM, HIV negative, African American | Peer led groupwork intervention based on Many Men Many Voices intervention protocol, addressing HIV/STI risk & building community. Weekly delivery over 6 sessions. | Race, Sexuality | Waitlist control | RCT | HIV risk behaviours, service access | N = 338  6 months  76% | Intervention participants had significantly greater reductions in unprotected anal intercourse, relative risk = 0.34 (95% CI = 0.14–0.83, P = 0.012). Intervention participants had 81% greater odds of testing for HIV (OR = 1.81, 95% CI = 1.08–3.01, P = 0.023). |
| 67 | Velasquez  2009  USA | GBMSM, HIV positive | An integrated, manualized intervention, using both individual counseling and peer group education/support. Four motivational interviews (therapist led) to promote alcohol abstinence and two days of group sessions (peer led) designed to promote safe sex. | HIV +, Sexuality | Control participants received resource referrals | RCT | Substance use, HIV risk behaviours | N = 253  12 months  70% | Participants in the control group drank 1.38 times the number of drinks per 30-day period, on average, than participants in the treatment group and had a higher number of heavy drinking days per 30-day period by a factor of 1.5. Reductions in unprotected sex across both intervention and control conditions. |
| 68 | Wolitski  2005  USA | GBMSM, HIV positive | Six 3-hour sessions of peer-led group intervention addressing relationships, HIV and STI transmission, drug and alcohol use, HIV status disclosure, and mental health. | HIV +, Sexuality | Standard HIV primary care, one session that provided safer sex information | RCT | HIV risk behaviours, STI incidence, psychosocial wellbeing | N =812  6 months  83% | Fewer intervention men reported unprotected sex with an HIV-seronegative or unknown-status partner compared with men in the standard intervention (21% vs 26%) effects were small, no other significant differences that were observed at both the 3 and 6-month follow-up assessments. |
| 62 | Harding  2004  UK | GBMSM | Nicotine replacement therapy and seven-week, peer led group work sessions, 2 hours each session. General population smoking cessation intervention repurposed for gay and bisexual men. | Sexuality | NA | Pre post | Substance use | N = 76  7 weeks  91% | Immediately post intervention, 44 out of 69 men were recorded as having quit smoking, this is a higher rate than general population evaluations of similar programs. |
