# Supplementary material for: Individual level peer interventions for gay and bisexual men who have sex with men between 2000 and 2020: A scoping review
Source: PLoS One. 2022 Jul 15;17(7):e0270649. doi: 10.1371/journal.pone.0270649 (PMC9286286; doi:10.1371/journal.pone.0270649)
Supplement: S3 Table — (DOCX) [file pone.0270649.s003.docx]

|  | | **Supplementary Table C: Peer navigation** | | | | | | | | |
| --- | --- | --- | --- | --- | --- | --- | --- | --- | --- | --- |
| **Ref #** | **Author**  **Year**  **County** | | **Population** | **Intervention** | **Peer Identity Characteristics** | **Comparison** | **Study type** | **Primary Outcomes** | **Sample**  **Follow Up**  **Retention** | **Effect Description** |
| 32 | Arayasirikul  2020  USA | | GBMSM, trans women, HIV positive, aged 18 – 34 | Text message intervention supporting people engaged in HIV care between appointments via care navigation, peer education, motivational interviewing, and social support. | Age, Gender, Sexuality, | NA | Pre post | Service adherence, viral suppression | N = 120  6 months  73% | HIV viral suppression increased over time among those who completed the intervention, 83.89% probability of viral suppression at 6 months vs 69.60% probability of viral suppression at baseline. |
| 28 | Graham  2020  Kenya | | GBMSM, HIV positive | Motivational interviewing intervention provided by trained providers, peers then provide wrap-around support to affirm pill taking, appointment reminders, and help to trace participants missing study visits. | +HIV status, Sexuality | Standard of care | RCT | Viral suppression, service adherence | N = 60  6 months  85% | The intervention group had a sixfold increased odds of viral suppression at follow up, this finding was significant despite a small sample size. |
| 76 | Reback  2019  USA | | GBMSM, trans women, HIV negative | Five session peer navigator programs designed to link transgender women and MSM to PrEP | Gender, - HIV status, Sexuality, PrEP use | NA | Pre post | Service access, service adherence, knowledge, and attitudes | N = 187  3 months  86% | 90% of trans women and MSM started PrEP, 80% of trans women and 70% of MSM reported they were still taking PrEP at follow up. Participants who elected to receive text reminders more likely to report PrEP adherence. |
| 75 | Cunningham  2018  USA | | HIV positive, GBMSM or trans women or heterosexual men | Twelve-session, twenty-four-week peer navigation intervention, encompassing counselling, goal setting and accompaniment to two HIV care visits. | +HIV status, Incarceration, Race, Substance use | Standard transitional case management | RCT | Viral suppression, service adherence | N = 356  12 months 70% | Viral suppression achieved by 49.6% of intervention participants comparted to 36.0% control. |
| 77 | Tanner  2018  USA | | GBMSM, transgender women, HIV positive | mHealth intervention on social media platforms to improve care engagement. Cyberhealth educators send theory informed messages to support health across the HIV care continuum. | Gender, Race, Sexuality | NA | Pre post | Service adherence, viral suppression | N = 91  1 year  82% | Significant reductions in missed HIV care appointments within 12 months (68.0% vs. 53.3%) and increases in viral load suppression (61.3% vs. 88.8%) at follow up. |
| 74 | Bouris  2017  USA | | GBMSM, HIV positive, African American, Aged 16 – 29 | Participants worked with interventionists to identify a support person already known to them, support person trained in intervention protocols to support retention in HIV care & ARV adherence. | Immediate friend, family or romantic partner | Standard of care | RCT | Service adherence, viral suppression | N = 106  12 months  91% | Intervention participants 3.01 times more likely to have had 3 HIV primary care visits in the previous 12 months. Self-reported ARV adherence also indicated that intervention participants were 2.91 times more likely to report ≥ 90% medication adherence. |
| 78 | Yan  2014  China | | GBMSM, HIV Positive | Peers provided one month of follow up support for those screened as HIV positive at a community-based point of care testing service. Support in the form of peer counselling and linkage to care. | Sexuality | Population health data | Pre post | Service access | N = 52  1 month  NA | 90% of those screened as HIV positive accessed HIV care within 1 month, this compared to 40% as recorded in all of population data sources. |
