# Supplementary material for: Individual level peer interventions for gay and bisexual men who have sex with men between 2000 and 2020: A scoping review
Source: PLoS One. 2022 Jul 15;17(7):e0270649. doi: 10.1371/journal.pone.0270649 (PMC9286286; doi:10.1371/journal.pone.0270649)
Supplement: S4 Table — (DOCX) [file pone.0270649.s004.docx]

|  | | **Supplementary Table D: Peer education** | | | | | | | | |
| --- | --- | --- | --- | --- | --- | --- | --- | --- | --- | --- |
| **Ref #** | **Author**  **Year**  **County** | | **Population** | **Intervention** | **Peer Identity Characteristics** | **Comparison** | **Study Type** | **Primary Outcomes** | **Sample**  **Follow Up**  **Retention** | **Effect Description** |
| 50 | Kelly  2020  USA | | GBMSM, HIV negative, African American, or multi-racial | Peers, identified as popular opinion leaders provided training to increase knowledge about PrEP, address PrEP concerns, endorse PrEP, and deliver education to peers. | HIV negative or unknown, Race, Sexuality | NA | Pre post | Service access, knowledge, attitudes | N = 40  3 months  89% | Significant increases in PrEP knowledge, attitudes, self-efficacy, and willingness to use PrEP. Participants reporting PrEP use increased from 3% to 11% |
| 31 | Patel  2020  India | | GBMSM, HIV negative | Peer-delivered, internet-based promotion of HIV testing and consistent condom use. Participants were assigned a peer outreach worker who they could communicate with via their chosen online platform. | Sexuality | Participants randomised to receive health promotion messages framed as either approach (desirable health outcome) or avoidant (prevent undesirable health outcome) | RCT | Service access, HIV risk behaviours, knowledge and attitudes | N = 244  6 months  52% | Significant increase in previous 6 months self-reported HIV testing from baseline to follow-up, 31.5% to 43.8%. Condom use during anal sex did not significantly change. No difference between avoidance and approach conditions. |
| 83 | Rhodes  2020  USA | | Latinx, GBMSM, trans women | 21 peers trained to provide education on HIV and STI testing, and condom use, and distribute safe sex supplies and health promotion resources among social network members. | Race, Sexuality | Waitlist control | RCT | Service access, HIV risk behaviours | N = 166  12 months  95% | At follow-up, participants reported increased HIV testing adjusted odds ratio 8.3 (95% CI 3.0–23.0). All study participants reported increased condom use; there was no significant difference between intervention and waitlist control participants. |
| 82 | Reback  2019  USA | | GBMSM, | Text message based intervention addressing methamphetamine use, three intervention arms. Arm 1, text messages & bi-directional interaction with peer educators. Arm 2, unidirectional, theory based gay specific messages plus auto weekly self- assessment. Arm 3, brief weekly text assessment of MA use and HIV. | Race, Sexuality, Substance use | Three interventions arms, as described in intervention column. | RCT | Substance use, HIV risk behaviours | N = 286  9 months  94% | All three conditions demonstrated reductions in methamphetamine use, sex while using methamphetamine, and condomless anal intercourse with casual male partners. Text message plus peer education intervention was associated with a mean reduction in previous 30 day, days of methamphetamine use from 11.4 at baseline to 7.8 at 9 months follow up. Overall automated delivery outperformed peer delivery. |
| 49 | Amirkhanian  2015  Russia / Hungary | | GBMSM | Peers identified as popular opinion leaders identified and trained to disseminate education around HIV risk reduction practices among social networks. | Sexuality | HIV testing and follow up counselling intervention | RCT | HIV risk behaviours, knowledge and attitudes, HIV/STI incidence | N = 586  1 year  90% | Proportion in intervention who engaged in any unprotected anal intercourse during the past 3 months declined from 54% at baseline to 43% at 12-month follow-up, proportion in comparison remained unchanged. Proportion who engaged in unprotected anal intercourse with a non-main sexual partner declined significantly more in intervention condition networks than comparison networks 18% to 9% vs. 23% to 21%. |
| 85 | Young  2015  Peru | | GBMSM, HIV negative | Peers identified as popular opinion leaders trained to have conversations with intervention recipients about HIV prevention and testing in private Facebook groups. | Sexuality | Private social media group with content regarding HIV testing and prevention but not peer delivered. | RCT | Service access, HIV risk behaviours | N = 556  3 months  90% | 43 participants (17%) in the intervention group and 16 (7%) in the control groups got tested for HIV, AOR 2.61 (95% CI 1.55–4.38). |
| 84 | Young  2013  USA | | GBMSM, HIV negative, African American and Latinx | Peer leaders trained to have conversations with intervention recipients about HIV prevention and testing in private Facebook groups. Participants could request a free, home-based HIV testing kit. | Race, Sexuality | Private social media group with content regarding HIV testing and prevention but not peer delivered. | RCT | Service access | N = 112  3 months  93% | Twenty-five of 57 intervention participants (44%) requested home-based HIV testing kits compared with 11 of 55 control participants (20%). Nine of the 25 intervention participants (36%) who requested the test mailed it back compared with 2 of the 11 control participants (18%) who requested a test. |
| 86 | Lau  2013  China | | GBMSM, HIV negative | Four peer educators identified at gay venues and by community organisations trained to deliver standardised and theory-based preventive messages to their peers via daily social interactions. | Sexuality | Access to general health and wellbeing resources. | RCT | HIV risk behaviours, knowledge and attitudes | N = 79  2 months  *No retention data* | Significant findings in relation to behavioural intentions to use condoms intervention 94.4% vs. control 60.6%, relative risk 1.56 (95% CI: 1.17, 2.08) and self-efficacy taking up HIV testing, intervention 97.2% vs control 72.7%, relative risk 1.34 (95% CI: 1.08, 1.66). |
| 47 | Amirkhanian  2005  Russia / Bulgaria | | GBMSM | Peer leaders sociometrically identified and trained to deliver ongoing theory-based HIV prevention advice to network members addressing AIDS risk-related knowledge and risk reduction norms, attitudes, intentions, and self-efficacy. | Sexuality | No intervention | RCT | HIV risk behaviours, knowledge and attitudes | 276  1 year  88% | Significant reductions in the percentage of experimental arm participants reporting unprotected intercourse with multiple partners 31.5% at baseline and 16.0% at 12 months follow up. Reductions among experimental arm participants with multiple partners, 76.0% at baseline and 57.3% at 12 months follow up. No significant reductions recorded in control group across these outcomes. |
| 48 | Amirkhanian  2003  Russia / Bulgaria | | GBMSM | Sociometric measures were used to identify social leaders who were trained to deliver theory-based and tailored HIV prevention conversations with peers. | Sexuality | NA | Pre post | HIV risk behaviours, knowledge and attitudes | 77  4 months  94% | Significant increases were found for all the theory-based mediators of behaviour change, these include knowledge or HIV risk reduction, attitudes towards condoms, perceptions of social norms about safe sex and self-efficacy for safe sex. There was a significant increase in levels of condom use with casual male partners between baseline and follow-up. |
